# Comparative Study of the Effects of Chamomile (*Matricaria Chamomilla *L.) and Cabergoline on Idiopathic Hyperprolactinemia: A Pilot Randomized Controlled Trial

**DOI:** 10.22037/ijpr.2019.1100758

**Published:** 2019

**Authors:** Marya Kabiri, Mohammad Kamalinejad, Soodabeh Bioos, Mamak Shariat, Farnaz Sohrabvand

**Affiliations:** a *School of Traditional Medicine, Tehran University of Medical Sciences, Tehran, Iran. *; b *School of Pharmacy, Shahid Beheshti University of Medical Sciences, Tehran, Iran. *; c *Institute of Family Health, Maternal, Fetal and Neonatal Research Center, Tehran University of Medical Sciences, Tehran, Iran.*; d *Vali-e-Asr Hospital, Imam Khomeini Hospital Complex, Tehran University of Medical Sciences, Tehran, Iran.*

**Keywords:** Hyperprolactinemia, Matricaria chamomilla, Cabergoline, Prolactin, Complementary medicine, Chamomile

## Abstract

Chamomile is a fascinating plant quoted in several traditional medicine texts, which has broad-spectrum pharmacological activity and medicinal uses. The aim of this study was to assess the efficacy of chamomile syrup in reducing serum prolactin in women with idiopathic hyperprolactinemia. The study was a randomized, controlled clinical trial that was conducted on 56 women with idiopathic hyperprolactinemia for a study period of four weeks. Patients were randomly enrolled in two parallel arms and were treated by chamomile syrup at a dose of 5 mL twice daily or cabergoline tablet orally at a dose of 0.25 mg twice weekly. Serum prolactin levels were measured at baseline and the end of the 4-week study period. Any report of adverse events was also recorded. Results revealed that within the cabergoline group the reduction in the mean prolactin level was significantly greater than that of the chamomile group (*p* <0.0001). It was also found that decline in the mean prolactin level was statistically significant within the chamomile group (*p* <0.0001). Chamomile syrup seems to be effective on serum prolactin reduction in women with idiopathic hyperprolactinemia. However, studies with a larger sample size and for a longer follow-up period are recommended.

## Introduction

An excessive rise in prolactin or hyperprolactinemia is a common endocrinological disorder in hypothalamic-pituitary region which affects the reproductive function in both sexes (1, 2). Prolactin synthesis and secretion is principally regulated by tonic inhibition of hypothalamic dopamine but several stimulating and inhibiting factors also contribute to this process. Prolactin plays a significant role in neuroendocrine-immune interactions (2, 3). Hyperprolactinemia may lead to clinical manifestation in both sexes or remain asymptomatic. Clinical manifestation in premenopausal women is usually irregular menses, amenorrhea, galactorrhea and infertility. Hyperprolactinemia may cause osteoporosis or osteopenia and progressive atherosclerosis (1, 2). Metabolic abnormalities such as hyperlipidemia, and hyperglycemia have been reported in people with hyperprolactinemia (4). Furthermore, the association between hyperprolactinemia and cardiovascular mortality (5) and some cancers have been found in recent studies (3, 6, 7). Sometimes, hyperprolactinemia is idiopathic when the recognizable etiologies cannot be found. In the long term, normalization of serum prolactin levels occurs in about 30% of patients with idiopathic hyperprolactinemia and a microprolactinoma appears in approximately 10% of them. Some individuals with idiopathic hyperprolactinemia may have a very small prolactinoma which cannot be detected by current imaging modalities (8). De Bellis *et al.* suggest an autoimmune involvement of pituitary in some cases of idiopathic hyperprolactinemia (9). Pharmacotherapy with dopamine agonists is recommended in symptomatic cases with idiopathic hyperprolactinemia. Dopamine agonist therapy can enhance biochemical and clinical response with high success in most patients of hyperprolactinemia. Cabergoline and bromocriptine as dopamine agonists are most frequently used. Among dopamine agonists, cabergoline has become the treatment of choice for cases with symptomatic idiopathic hyperprolactinemia owing to its long half-life, high effectiveness and better tolerability. Limited data regarding the safety of cabergoline in pregnancy and its high cost are disadvantages of this dopamine agonist compared with bromocriptine (1, 3, 8). In spite of the excellent performance of dopamine agonists, these drugs may have some adverse reactions in the gastrointestinal, nervous and cardiovascular systems (1, 8). Furthermore, dopamine agonist resistance and recurrence of hyperprolactinemia after dopamine agonist withdrawal occur in some patients, although recurrence of idiopathic hyperprolactinemia after dopamine agonist withdrawal is lower than both micro- and macroprolactinomas (1, 10). Numerous studies have reported improvement in lipid profile and reduction of fasting plasma glucose in hyperprolactinemic patients and improvement in glucose metabolism in prediabetes cases after treatment with cabergoline (4, 11). 

Currently, people are more willing to use complementary and alternative medicine (CAM) all over the world (12, 13). Several studies have indicated the efficacy of herbal remedies in the traditional management of hyperprolactinemia (14, 15). On the other hand, recent studies have shown the valuable role of natural products in drug discovery which leads to progress in the treatment of human ailments (16).

Chamomile (syn. *Chamomilla recutita *L., *Matricaria chamomilla* L.) is a fascinating, well-known, widely used and well-documented medicinal plant and it has been mentioned by Hippocrates, Dioscorides, Galen, Avicenna and other prominent scholars from the history of medicine (17-19). Studies have shown that chamomile contains the following phytochemical compounds: flavonoids and other phenolic compounds, essential oil, polysaccharides, amino acids, fatty acids and mineral elements (20, 21). These different classes of bioactive components play essential biological roles such as antioxidant activity, anti-cancer activity, anti-inflammatory activity and osteoporosis prevention (22-25). Studies with animal models suggest its potency as an antidiabetic and hypocholesterolemic herbal medicine (26). Zand *et al*. found that chamomile extract at the highest concentration tested has a weak estrogenic and progestational effects (27). Previous studies have shown that some flavonoid components of chamomile possess different pharmacological effects on the central nervous system by interacting with neurotransmitter and neural system, like GABAergic, serotonergic, noradrenergic and dopaminergic which may produce sedative, anxiolytic and antidepressive properties (28-30). The clinical efficacy of chamomile on some conditions relevant to menstruation including dysmenorrhea, PMS and mastalgia, which is related to premenstrual syndrome, have also been demonstrated (31, 32).

Based on Persian traditional healing science dating back to thousands of years, chamomile has various medicinal properties (17, 33). Avicenna in his writings refers to chamomile as one of the medicinal herbs that induce menstrual bleeding and he recommends it for amenorrhea or oligomenorrhea (17). Famous medieval Persian scholars believed that medications which can induce menstrual bleeding are useful for treating women with galactorrhea alongside amenorrhea or oligomenorrhea (34). Avicenna also believed that chamomile has beneficial effects on the brain and nervous system. In Persian traditional medicine texts, the anti-inflammatory and diuretic effects of chamomile have been reported (17).

According to some pharmacological activities of chamomile such as interaction with neurotransmitter and neural system (28, 29) and its antioxidant (21), anticancer (24) and anti-inflammatory effects (23), along with some of its medical and traditional uses, we decided to investigate the efficacy of chamomile syrup in reducing prolactin levels and compare it with that of cabergoline by conducting a 4-week, pilot, randomized, controlled clinical trial in women with idiopathic hyperprolactinemia.

## Experimental

*Study design*

This was a pilot randomized clinical trial study with two parallel interventional arms (i.e., chamomile syrup and cabergoline tablet groups) and a 1:1 allocation ratio. Institutional review board of Tehran University of Medical Sciences (TUMS) approved the study proposal with reference number 93/d/130/45. Then the trial was recorded in the Iranian Registry of Clinical Trials (IRCT) with reference number IRCT2013122215891N1. All eligible women signed an informed consent after notification of research process and before inclusion in the study.

**Figure 1:**
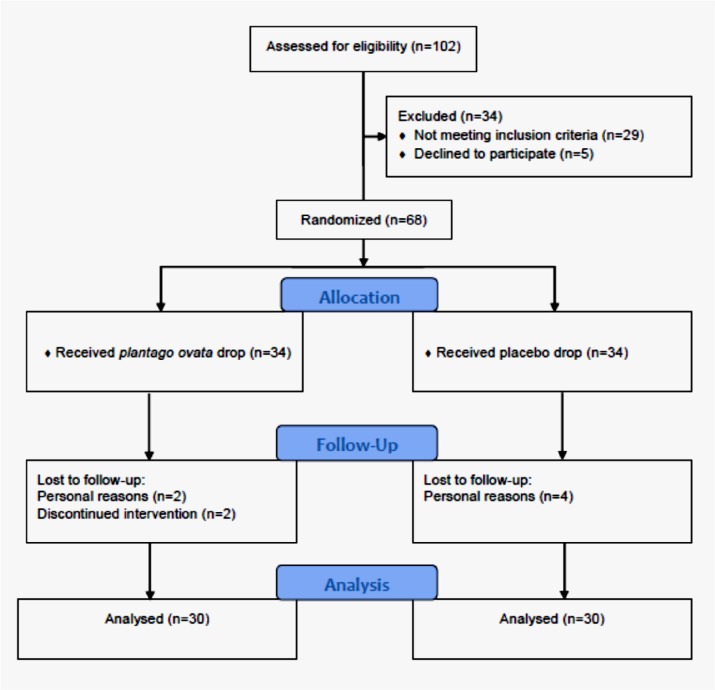
The CONSORT flowchart of the study

**Table 1:** The baseline clinical characteristics of the women with idiopathic hyperprolactinemia in the chamomile and the cabergoline groups

**Variable**	**Chamomile group (n = 28)**	**Cabergoline group(n = 28)**	***p*** **-value**
Age (years), Mean (± SD)	27.75 (± 4.94)	27.32 (± 4.72)	0.74
BMI (kg/m2), Mean (± SD)	24.16 (± 3.86)	23.48 (± 3.14)	0.47
Baseline serum prolactin (ng/mL), Mean (± SD)	38.20 (± 12.44)	40.12 (± 14.36)	0.92

SD: standard deviation, BMI: body mass index.

**Table 2:** Comparing serum prolactin values of the women with idiopathic hyperprolactinemia who were treated with chamomile syrup or cabergoline before and after four weeks of intervention

**Before intervention prolactin (ng/mL)**		**After intervention prolactin (ng/mL)**	***p*** **-value**
Chamomile	38.98 ± 12.95	22.99 ± 14.73	< 0.0001
Cabergoline	40.12 ± 14.36	10.98 ± 12.20	< 0.0001
*p*-value	0.957	< 0.0001	

Prolactin values are expressed as mean ± SD.

*Preparation of the materials*

The chamomile flowers were collected from Dezful (Khuzestan province) located-in the southwest of Iran in May 2014. The sample was authorized and kept at the herbarium of Shahid Beheshti University of Medical Sciences with voucher no.8060 – sbmu.

Aqueous extract of chamomile flower was prepared according to traditional methods (35). One hundred grams of dried chamomile flowers was placed in a beaker and one liter of water was added. The mixture was boiled on the heater for 10 min and was left for around 4 h in the laboratory. Then the contents of the beaker were filtered and condensed by using the bain-marie. Finally, 20 g of dry extract was obtained from 100 g flowers of chamomile. Then a syrup containing 10% of the extract was made with USP method (Sugar 66.7% w/w) and was poured into bottles of 200 mL.

*Standardization of chamomile syrup*

The total phenolic content of chamomile syrup was determined by the Folin–Ciocalteu method (36) and the total flavonoid content of chamomile syrup was determined by the aluminium chloride method (37). Total phenolic content as gallic acid equivalent per 1 mL of chamomile syrup, was 3.172 mg and total flavonoid content as rutin equivalent per 1 mL of chamomile syrup, was 1.376 mg.

*Participants*

Women patients referred to the obstetrics and gynecology outpatient clinic of the Imam Khomeini hospital affiliated to the Tehran University of Medical Sciences (TUMS) with idiopathic hyperprolactinemia aged 18-45 yrs. were enrolled in the study if their serum prolactin values were greater than 25 ng/mL. Patients with amenorrhea, underlying disease including diabetes, hypertension, cancer and a history of hyperprolactinemia treatment in the last 3 months were excluded from the study. Patients with a history of sensitivity to ergot derivatives, hay fever and allergic reaction to herbals of the *Compositae* family were also excluded. Another exclusion criterion was consumption of warfarin. None of the participants had a history of alcohol usage and none of them were smoker. Whenever pregnancy was suspected or planned, the intervention was stopped. 

Demographic characteristic, medical history, physical examination and biochemical laboratory tests, and symptoms related to hyperprolactinemia of the participant were also determined and recorded.

*Diagnosis of idiopathic hyperprolactinemia*

When increased prolactin level in the initially evaluated female patients was found, the assessment to exclude secondary causes of hyperprolactinemia was performed by a gynecologist. Idiopathic symptomatic hyperprolactinemia was diagnosed via a detailed medical history, physical and gynecological examination and laboratory analysis. Hyperprolactinemia related to physiological or pathological causes such as pregnancy, breastfeeding, thyroid disorders, liver failure disease, kidney failure disease, adrenal disorders and hypothalamic pituitary dysfunction was appropriately investigated. Moreover, a review of medications used by patients such as antipsychotics, antidepressants, opiates, metoclopramide, domperidone, estrogen, verapamil, reserpine, cimetidine, ranitidine and any medications and herbal agents that could be effective on prolactin secretion, in the past three months was carried out. Patients on these drugs were excluded from the study. 

Biochemical measurements such as thyroid, hepatic and kidney function parameters, blood levels of LH, FSH, and pregnancy test were assessed for all patients. If applicable, a magnetic resonance imaging (MRI) of the pituitary region was performed to roll out pituitary disorders. Women with idiopathic hyperprolactinemia had normal biochemical, and imaging findings. 

*Interventions*

Eligible patients were randomly assigned to receive either chamomile syrup at a dose of 5 mL, twice daily, after meal (intervention group), or cabergoline tablets (Caberlin^®^, manufactured by Iran Hormone Pharmaceutical, Iran) at a dose of 0.25 mg twice weekly (control group), for a period of four weeks. 

*Outcomes*

The serum prolactin value was the primary outcome measure. In both groups, incidence of possible allergic or adverse reactions to the study drugs was watched and recorded.

*Hormone Assays*

Serum prolactin levels were measured by the immunoradiometric assays (IRMA) kits (manufactured by Padyab Teb, Iran). The normal value of serum prolactin was (2-22 ng/mL) for females. In order to determine serum prolactin levels, blood samples were collected from participants after an overnight fasting within two hours after awakening.

*Safety measures*

All participants in both groups were assessed for four weeks of the study period. Patients were asked to report any possible adverse reactions to the physician in charge by phone call. In addition, the follow-up visits were performed to evaluate drug intake, concomitant medication usage, compliance and any complications two and four weeks after the receipt of interventions. A checklist was applied to record any possible adverse reactions. Pregnancy test was carried out in participants who had a delay in menstruation. 

*Randomization, Allocation Concealment and Blinding*

Allocation of participants in each intervention arms (i.e., chamomile or cabergoline) was carried out using a block-randomization list with equal length blocks (size of 4), which were non-stratified and were generated by Excel software. The allocation sequence was concealed by generating unknown codes. The participants and researchers were not blind to the interventions because of the dosage form of the interventions *i.e.* syrup and tablet. Statistician was blind to the intervention and control groups.

*Statistical Methods, analysis*

Data were analyzed with the Statistical Package for the Social Sciences (IBM^®^ Corporation version 20) software. The continuous variables were expressed as means ± SD. According to Kolmogorov-Smirnov test, serum prolactin values were not normally distributed, therefore between groups comparisons were performed using the Mann-Whitney U test. Also, the Wilcoxon test was applied for within group analysis of the changes in prolactin levels. In addition, the Chi-square test was used to compare the response rate in two groups.

## Results

From July 2014 to August 2016, a total of 73 female patients were assessed for inclusion and exclusion criteria, of which 56 patients met the study criteria. 25 participants in the chamomile group and 27 participants in the cabergoline group completed the study and underwent data analysis. Study flowchart is presented in Figure 1.

Table 1 shows the baseline clinical characteristics of women in the study groups. There were no significant differences between age, body mass index and baseline serum prolactin values of the two groups.

The mean ± SD values of the prolactin levels in the study groups, before and after four weeks of intervention, were shown in table 2. 

The Wilcoxon test showed that there was a significant decrease in the plasma levels of prolactin in the cabergoline group (*p* <0.0001). Similarly, prolactin levels in the chamomile group was reduced significantly (*p* <0.0001). 

In addition, at the end of the four weeks of study period, the mean prolactin levels in the cabergoline group was significantly lower than that of the chamomile group (*p* <0.0001) 

(table 2).

Between groups comparison of the number of patients with a reduction in the prolactin levels showed that the prolactin normalization rate in the cabergoline group was higher than that of the chamomile group (96% versus 72%) (*p* = 0.013).

Regarding the reported adverse reactions, two patients receiving cabergoline (7.1%) reported drug- related postural hypotension and nausea. In one of the patients with postural hypotension, the symptom was resolved by reducing the dose of the cabergoline within 3 weeks, however the patient complaining of nausea dropped out of the study. In the chamomile group, there was no report of any adverse reaction. The incidence of complications in the two study groups were not statistically significant (*p* = 0.175).

## Discussion

This study presents the first pilot, randomized, controlled trial of chamomile syrup versus cabergoline in reducing prolactin levels in women with idiopathic hyperprolactinemia. The results indicated that the mean serum prolactin levels were decreased in the both cabergoline and chamomile groups after four weeks treatment, nevertheless, cabergoline was more effective than chamomile syrup in reducing serum prolactin levels. 

Chamomile is one of the fascinating medicinal plants which has broad-spectrum of pharmacological activity and medicinal uses (18, 21). It is notable that cabergoline is considered as a treatment of choice due to its efficacy and tolerability in the majority of people with symptomatic idiopathic hyperprolactinemia (1, 8).

The precise mechanism of prolactin reduction effect by chamomile is not clear. Nonetheless, there would be several plausible mechanisms responsible for biological properties of the chamomile that can eventually lead to reduction in prolactin levels. Sharaibi *et al.* investigated ethnobotanical and phytochemical properties of 13 indigenous medicinal plants used for the traditional treatment of women with hyperprolactinemia in Lagos State, Nigeria. Their findings demonstrated presence of several important bioactive components such as alkaloids, phenols, tannins, flavanols, flavonoids, steroids, saponins, proanthocyanidins, anthraquinones and cardiac glycosides in these plants. The presence of flavonoids and phenolic compounds was reported in all of the 13 medicinal plants which are effective in the treatment of hyperprolactinemia. The presence of tannin in all the plants except two of them was also confirmed (14). Other phytochemical studies have also indicated the existence of phenolic compounds and a variety of flavonoids as well as tannins in chamomile (38, 39).

Studies have shown that natural phenolic compounds possess a wide range of biological activities of anti-inﬂammatory, anticancer, antimutagenic and antioxidant effects. These effects contribute to chemopreventive potential of phenolic compounds (40). According to the results of several studies, chamomile has a potent antioxidant (22) as well as anti-cancer property (24). Studies have suggested that apigenin, a major flavonoid isolated from chamomile, has considerable potential to prevent cancer. The ability of apigenin to inhibit the growth of a wide variety of human cancer cells such as colon cancer, breast cancer, prostate cancer, neuroblastoma, leukemia, skin cancer and thyroid cancer has been demonstrated (41). Anti-inflammatory property of chamomile has been known for centuries. Recently, many studies have also demonstrated the anti-inflammatory properties of chamomile (23, 42). Apigenin, chamazulene and alpha-bisabolol are the most important anti-inflammatory components of chamomile. Laboratory studies have shown that apigenin inhibits pro-inflammatory agents (20, 21, 23). Chamazulene may play an important role in autoimmune disorders such as rheumatoid arthritis, inflammatory bowel disease and psoriasis (21) and it has been reported that another flavonoid, luteolin may be effective in the treatment of autoimmune diseases (43). Luteolin is one of the major flavonoids that exists in chamomile (20). It has been reported that about 10% of cases of idiopathic hyperprolactinemia will develop microadenoma and in some cases, an autoimmune involvement of pituitary has been found. De Bellis *et al.* found antipituitary antibodies in 25.7% of patients with idiopathic hyperprolactinemia. They also observed a partial anterior pituitary impairment in 35.3% of patients with positive antipituitary antibodies (8, 9). These suggest that antioxidant, anti-cancer and anti-inflammatory properties of chamomile may be effective in the treatment of idiopathic hyperprolactinemia.

Studies have shown that there is a link between premenstrual symptoms especially mastalgia and latent hyperprolactinemia (44-46). In a study by Sharifi *et al.* on 90 patients, effectiveness of chamomile extract and mefenamic acid on PMS severity was compared. They found that the overall intensity of somatic and particularly psychological premenstrual symptoms in chamomile group decreased more than those in the mefenamic acid group (31). Sharifi *et al.* also revealed that chamomile extract and mefenamic acid could improve premenstrual mastalgia intensity and severity without significant difference between two groups (32). One interesting possibility is that chamomile may be able to suppress the secretion of prolactin, which improves the severity of PMS and breast pain in a similar manner as bromocriptine and *Vitex agnus-castus* (chaste tree) do. Previous studies have shown the relationship between reducing serum prolactin level and ameliorating premenstrual mastalgia with bromocriptine as a synthetic dopaminergic agent (45) and several scientific studies support the efficacy of *Vitex *extracts in the management of PMS and cyclical mastalgia (47, 48). *Vitex* exhibits central dopaminergic activity and suppresses prolactin secretion in preclinical and clinical experiments (46, 48, 49). Kilicdag *et al.* have investigated the efficacy of *Vitex *compared with bromocriptine for the reduction of serum prolactin level in 40 women with mild hyperprolactinemia and also ameliorating mastalgia in 40 women with cyclic mastalgia in a randomized clinical trial study of three months duration. According to their results, no significant difference was found between *Vitex* and bromocriptine groups in reducing serum prolactin levels and ameliorating breast pain (48). It seems dopaminergic effects of some *Vitex *compounds that reduce serum prolactin level are mechanisms to control PMS and mastalgia (46, 48).

In an animal model, Nakazawa *et al.* suggest that the dopaminergic system may interfere with the antidepressant activities of apigenin in the mouse brain. Their study also showed that apigenin is more speciﬁc towards the hypothalamus and amygdala (30). It is noteworthy that apigenin is a major flavonoid available in chamomile (20).

Results from the above studies confirm our findings and support the hypothesis that chamomile can inhibit prolactin secretion.

Postural hypotension and nausea as the adverse events of cabergoline consumption were also reported in prior studies (50). Although in our study, the difference between the rate of the adverse events in two groups was not statistically significant, no adverse event was seen in the chamomile group. 

It should be acknowledged that precise evaluation of the subjective and objective improvement of patients with idiopathic hyperprolactinemia requires a long-term study period. A long-term study may lead to a better judgment on the effects of the study drugs on the serum prolactin, clinical symptoms, as well as their safety and tolerability. Moreover, other limitations of this study including small sample size, using only a fixed dose of chamomile and the open-label study design should be contemplated in the interpretation of the findings.

## Conclusion

Chamomile syrup seems to be effective on serum prolactin reduction in women with idiopathic hyperprolactinemia. Further studies with a larger sample size, long-term intervention period, long-term follow-ups and simultaneous assessment of the symptoms are strongly recommended. Moreover, preclinical studies to investigate the precise mechanisms and effects of chamomile in animal models are recommended. 
